# Influences of heart rate feedback and autistic traits on affective mindreading

**DOI:** 10.1038/s41598-024-69038-7

**Published:** 2024-08-13

**Authors:** Katharina Bögl, Mareike Bayer, Henrik Walter, Isabel Dziobek

**Affiliations:** 1 Clinical Psychology of Social Interaction, Department of Psychology, Faculty of Life Sciences, Humboldt-Universität zu Berlin, Berlin, Germany; 2 Berlin School of Mind and Brain, Humboldt-Universität zu Berlin, Berlin, Germany; 3 German Center for Mental Health (DZPG), partner site Berlin, Berlin, Germany; 4 Department of Psychiatry and Psychotherapy, Charité Universitätsmedizin Berlin, corporate member of FU Berlin and Humboldt-Universität zu Berlin, Berlin, Germany

**Keywords:** Human behaviour, Social behaviour

## Abstract

Although mindreading is an important prerequisite for successful social interactions, the underlying mechanisms are still matter of debate. It is unclear, for example, if inferring others’ and own mental states are distinct processes or are based on a common mechanism. Using an affect-induction experimental set-up with an acoustic heart rate feedback that addresses affective mindreading in self and others, we investigated if non-autistic study participants relied on similar information for self- and other-directed mindreading. We assumed that due to altered mindreading capacities in autism, mainly individuals with low autistic traits would focus on additional sensory cues, such as heart rate, to infer their own and their gambling partner’s affective states. Our analyses showed that the interpretation of a heart rate signal differed in self- and other-directed mindreading trials. This effect was modulated by autistic traits suggesting that individuals with higher autistic traits might not have interpreted the heart rate feedback for gambling partner ratings and differentiated less between self- and other-directed mindreading trials. We discuss these results in the context of a common mechanism underlying self- and other-directed mindreading and hypothesize that the weighting of internal and external sensory information might contribute to how we make sense of our and others’ mental states.

## Introduction

Inferring others’ mental states, also referred to as “mindreading” or “having a theory of mind”^1^, is a crucial ability towards the understanding and prediction of the behavior of others^1^. Depending on the sort of inference, one can distinguish cognitive and affective Theory of Mind^2^, whereby inferences about feelings of others can be considered the latter^3^. In the last decades, different theories about the cognitive and neural mechanisms underlying mindreading emerged. Some theories argue that the underlying mechanism to mindreading is the same when inferring own or others’ mental states^4^. The premise of only one common underlying mechanism to self- and other-directed mindreading is the core principle of so called one-mechanism theories.

An auspicious candidate of one-mechanism theories is the *interpretive sensory-access (ISA) account *^4^ which claims that the access to propositional attitudes (like deciding or judging) takes place via a mindreading faculty that interprets globally broadcast perceptual information. According to this account, the mindreading faculty originally evolved to understand other’s minds. Therefore, the same sensory channels that are used when inferring mental states of others (e.g., observing others’ behavior) can be used to infer own mental states (e.g., observing own behavior). In principle, all forms of globally broadcast representation can be interpreted by the mindreading faculty, thus also imagistic representations like inner speech or visual imagery will be used to infer own mental states^4^. Taken together, while access to own sensory signals is direct, access to own mental states is supposed to be an interpretation of globally broadcast information and thus indirect^5^. Consequently, humans can not only be mistaken about other’s but also about their own mental states. Previous studies have indeed demonstrated this fallibility of first-person access to own mental states: For instance, while listening to the content of a simulated radio broadcast, participants that were told to nod the head would agree more to the statements as compared to participants that were told to shake their head^6^. This fallibility is of high relevance especially in psychiatry where misinterpretations of behavior or sensory states can manifest in psychiatric disorders (e.g., panic disorder). More recent research for instance showed that manipulated sensory feedback can affect first person judgments^7–9^ and favors the idea that humans interpret sensory information equivalently when judging one’s own or others’ mental states.

If indeed one common mechanism underlies mindreading in self and others, then individual differences between participants in mindreading of others should also manifest in mindreading the self. A prominent clinical candidate with regard to alterations in mindreading of others is autism. Autism is defined through impairments in social communication and interaction, co-occuring with patterns of repetitive, stereotyped behaviors and activities^10^. Recent studies estimate the prevalence of autism at approximately one to two percent in the general population^11,12^. In the search for a causal account of the symptomatology associated with autism, the notion of a Theory of Mind deficit in autism emerged^1^, and many studies have indeed reported altered mindreading of others in autism^13–17^. However, these differences in mindreading did not explain the full range of phenomena associated with this condition, like aberrant sensory processing^18,19^. Therefore, mindreading alterations are nowadays seen as a possible manifestation of difficulties in social interaction and communication rather than the causal mechanism underlying autism symptomatology. In the last years, researchers set an additional focus on the inference of own mental states in autism^14,20^. The results of these studies indicate that not only mindreading of others but also mindreading the self might be different in autism. Clearly, these results speak for the idea of a common underlying mechanism to reading mental states of others and the self.

So far, only few studies compared self- and other-directed mindreading (e.g.,^21–24^), particularly under the notion of one-mechanism theories. A potential reason for this is the paucity of suitable experimental designs that assure that self and other conditions contain identical information. Otherwise, differences in self- and other-directed mindreading might be attributable to different experimental cues. To overcome this potential confound, we opted for inducing current mental states in identical self and other conditions. We therefore used a gambling paradigm in which two participants were asked to constantly infer their own or their gambling partner’s current affective mental state. If one-mechanism theories hold true, similar judgements should occur when identical internal and external information during self-and other-directed mindreading are presented.

Our set-up was inspired by studies investigating cognitive processes underlying emotion attribution in self and others using a wheel of fortune gamble^25,26^. To evoke positive and negative affective states, we presented wheels of fortune in different gambling outcomes (winning, loosing or a neutral condition). The wheels were composed of three segments, each indicating one of the three different outcome possibilities. The wheels also varied within the size of the segments to incorporate different expectancy values to make the set-up more engaging for participants. Building on the ISA account^4^, we were further interested if affective mindreading in self and others relies on an interpretation of sensory information. According to the ISA theory, the mindreading faculty relies on the same sensory channels that are used to infer others’ minds when inferring own mental states. We therefore wanted to use a sensory signal that could, in principle, be interpreted via the same channel when inferring own or another persons’ mental states. We chose to feedback an acoustic signal representing the heart rate (HR) of the participant and their gambling partner, respectively (self vs other condition), as this operationalization was used in previous literature^27–29^. The signal was presented in a control and, unknown to the participants, an accelerated condition, to investigate if the signal would be used to infer own and others’ affective mental states similarly. We expected the fastened HR signal to increase the reported affective judgements, meaning that during accelerated HR feedback trials, negative outcomes should be rated as more negative and positive outcomes as more positive. We based this assumption on previous research showing that an accelerated HR feedback intensified the perceived emotional intensity of neutral face stimuli^27^. Further, we were interested in the role of autistic traits in self- and other-directed affective mindreading. Notably, also non-autistic individuals present different levels of autistic traits^30^ (as measured with the Autism-Spectrum Quotient AQ), with meaningful effects on mindreading^31–33^. Prior research also showed that especially ambiguous stimuli in mindreading tasks are difficult to interpret for autistic participants^34^ (also see meta-analysis by^35^). Therefore, we expected to find an effect of autistic traits regarding the interpretation of ambiguous information, such as the accelerated HR signal. More specifically, we assumed that individuals with higher autistic traits would incorporate the HR feedback less when judging their own or their gambling partner’s mental states compared to individuals with lower autistic traits. Regarding the interpretation of unequivocal information, like the outcome of each gambling trial, we did not expect to find any differences between participants in dependence of their autistic trait levels.

Building on the presented literature, we derived the following three hypotheses: First, due to the identical set-up of our self and other conditions, we expected that the experimental information (i.e., the gambling outcome, the expectancy value, and the HR signal) would be interpreted similarly in self- and other-directed affective mindreading trials. Thus, no differences between self- and other-related judgements should arise. Second, we assumed that self- and other-directed affective mindreading would both be indirect and rely on the interpretation of sensory information (i.e., the HR feedback) and thus change between the HR manipulations. Third, we hypothesized that individuals with higher autistic traits would use the ambiguous information, i.e., the HR feedback, to a lesser extent than individuals with low autistic traits in self and other conditions.

## Results

### Model comparisons: different information used in self- and other-directed affective mindreading

To unravel if the experimental information was interpreted similarly in self- and other-directed affective mindreading trials, we conducted moderation analyses and compared the different models using AIC, BIC, and likelihood ratio tests. Our model comparisons revealed that our data was described best by adding two moderation effects that included the factor Target Person (self vs other). The moderation effects indicate that the experimental information (gambling outcome, expectancy value of the wheels, and the HR manipulation) had different effects in self- and other-related affective judgements. This finding therefore contradicts our first hypothesis concerning identical processes in self- and other-directed affective mindreading. The final model included the significant predictors *Outcome*, *Expectancy Value*, HR Feedback*Target Person, and HR Feedback*Target Person*AQ. Below, we will describe the different predictors of this model in the context of our hypotheses.

### Manipulation check: ratings depend on outcome and expectancy value

**Figure 1:**
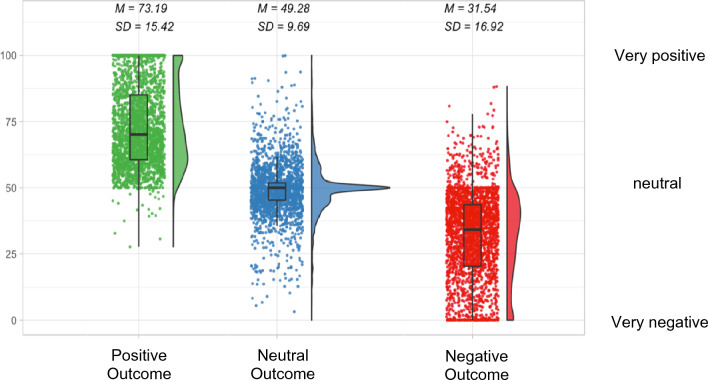
Mean ratings and rating distributions for the different Outcome conditions. *Note.* Mean ratings are displayed across all HR Feedback and Target Person conditions.

As expected, the factor Outcome displayed a significant effect: Our results showed that positive (beta = 21.70, *p* < .001) and negative gambling outcomes (beta = − 14.73, *p* < .001) were significantly related to the affective ratings. The neutral outcome condition was used as reference condition represented in the intercept, thus no specific beta coefficient for this factor is reported. Results of post-hoc paired *t*-tests between the different Outcome conditions indicate that all outcomes were rated significantly different (positive vs neutral outcome: *t*(2351) = − 66.2, *p* < .001; negative vs neutral outcome: *t*(2350) = 50.7, *p* < .001; negative vs positive outcome: *t*(2351) = 70.0, *p* < .001). We interpret these findings in favor of our experimental manipulation, showing that the different gambling outcomes induced different affective states (i.e., winning trials were rated with a positive and losing trials with a negative valence). Means and standard deviations for the different outcomes are depicted in Fig. 1.

In addition to the significant effect of the different Outcome levels, we also found a significant effect of Expectancy Value (beta = − 0.95, *p* = .001). In line with our expectations, Expectancy Value and affective ratings were significantly negatively related, i.e., when participants lost money, trials with higher expectancy values were rated as more negative because participants had higher expectations to win.

### Differential effects for self- and other-directed affective mindreading

As explained above, the model comparisons revealed that our data was described best by adding two moderation effects that included the factor Target Person (see supplement for all model specifications and indices). First, adding a moderation effect to the effect of HR Feedback improved model fit (AIC_without_interaction_: 52034.5 vs AIC_with_interaction_: 52032.55; BIC_without_interaction_ : 52220.03 vs BIC_with_interaction_: 52224.95; delta likelihood ratio = 3.95, *df* = 1, *p* = .047). The fit was further improved by adding a moderation effect for the interaction of HR Feedback and AQ (AIC_without_interaction_: 52032.55 vs AIC_with_interaction_: 52029.46; BIC_without_interaction_: 52224.95 vs BIC_with_interaction_: 52235.61; delta likelihood ratio = 7.09, *df* = 2, *p* = .029).

The two moderation effects argue against identical processes in self- and other-directed affective mindreading and can thus be interpreted as evidence against our first hypothesis.

### No modulation of Outcome through HR Feedback

Our second hypothesis was that mindreading in self and others is an indirect process and relies on the interpretation of sensory information. Therefore, we assumed that the accelerated HR signal would intensify affective ratings in a way that losing trials would be rated as more negative and winning trials as more positive. Consequently, we expected an interaction effect of Outcome and HR Feedback. Our results show that the interaction effect was neither significant for negative (beta_negative/accelerated_ = 0.48, *p* = .69) nor positive (beta_positive/accelerated_ = 0.01, *p* = .996) Outcome trials. Thus, contradicting our second hypothesis, we could not demonstrate that affective ratings for self and others were intensified through the interpretation of sensory information.

### Different use of HR Feedback for self and other ratings

**Figure 2:**
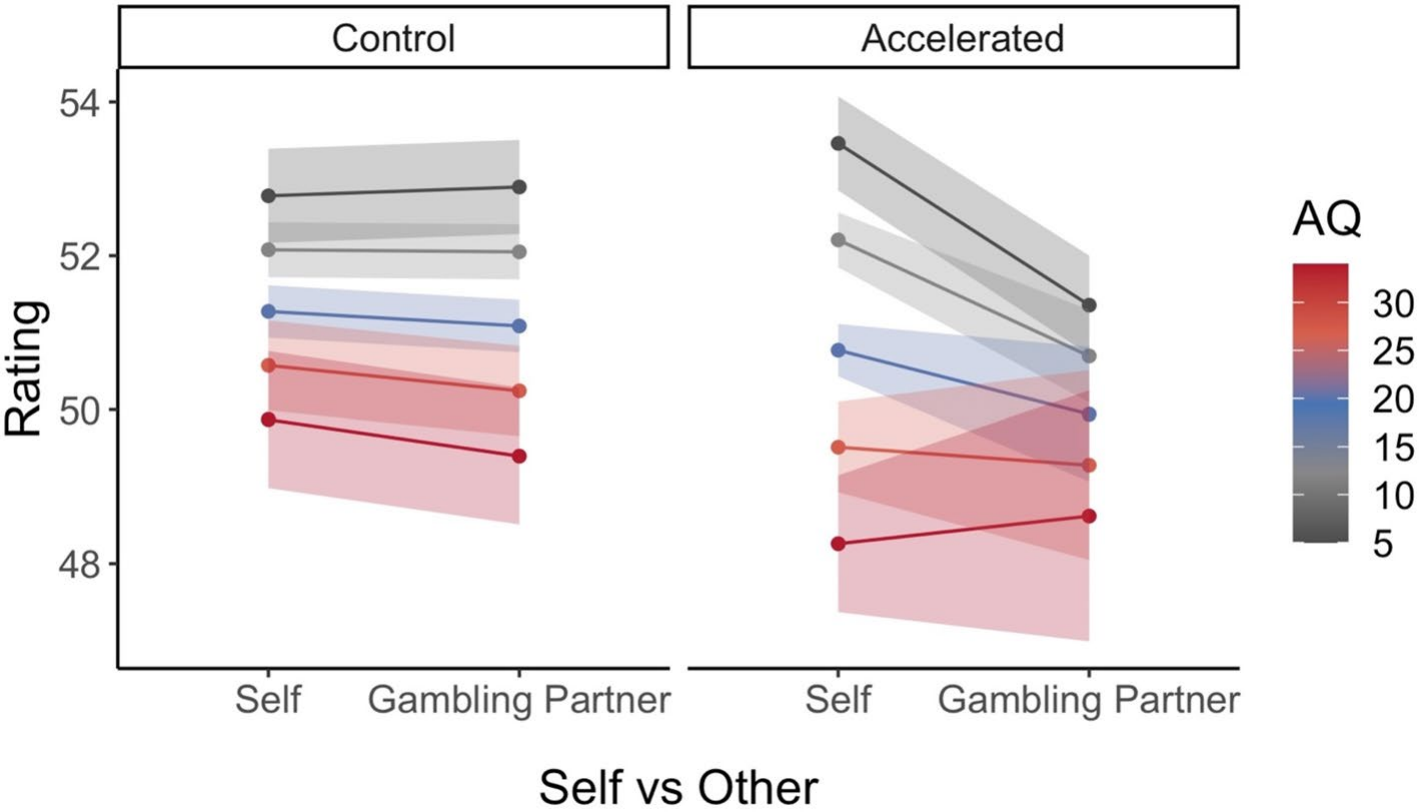
Estimated effects of the three-way interaction HR Feedback*Target Person*AQ *Note.* The left panel shows estimated effects averaged for all Outcome conditions during the control HR Feedback—divided for self and other trials. The right panel displays the effects during accelerated HR Feedback trials. The colored lines represent the estimated effects for the different levels of autistic traits with the respective standard errors being displayed as shaded areas.

However, our analyses revealed an interaction effect of Target Person and HR Feedback. The model estimate suggests that ratings for the gambling partner (other) showed lower values (i.e. more negative affective states) than self ratings in accelerated feedback trials (beta_accelerated/other_ = −2.75, *p* < .01; see Fig. 2). Two conclusions can be derived from this finding: First, participants used the sensory signal only for other-, and not for self-related ratings. A visualization of this effect can be seen in the right panel of Fig. 2 (especially for low autistic traits depicted in gray). Second, the accelerated HR Feedback did not intensify positive and negative outcomes but led to more negative ratings across all Outcome conditions.

### The role of autistic traits

Since we could not verify our second hypothesis, namely that accelerated HR Feedback would intensify affective ratings, we could also not verify our third hypothesis that autistic traits would alter this specific association. Nevertheless, autistic traits showed a significant moderation effect: The interaction effect of HR Feedback and Target Person was related to the individual AQ scores as suggested through a significant three-way interaction. The corresponding regression coefficient indicates that participants with higher AQ scores rated their gambling partner as having fewer negative feelings in the accelerated HR Feedback condition than participants with lower AQ scores (beta_accelerated/other/AQ_ = 0.105, *p* < .01). It seems that mostly participants with low autistic traits differentiated between self and other-related affective ratings in the accelerated HR Feedback condition (see Fig. 2). With increasing AQ scores (depicted in red), this differentiation decreased.

## Discussion

Using our self-other gambling set-up, we were able to induce different affective mental states in non-autistic study participants, who also attributed these mental states to their gambling partner in response to the same stimuli. In addition to the main effect of gambling outcome, we were also able to find a significant relation between ratings and the theoretical expectancy values. In contrast to our first hypothesis, our results indicate that the processes during self- and other-directed affective mindreading, despite the largely identical setup, were not the same. Statistically, this finding is based on our model comparisons that showed that the best fitting model incorporated two moderation effects. The moderation effects demonstrate that the relations between experimental information, i.e., HR feedback, and affective ratings were different for self and other ratings. Also, in contrast to our second hypothesis, accelerated HR feedback did not intensify affective ratings in self and other trials. Yet, we found that during accelerated HR feedback trials, the affective state of the gambling partner was rated as more negative as compared to self ratings. This result suggests that participants used the HR feedback to judge the affective state of their gambling partner. As we had to reject that outcome ratings would be intensified through the HR feedback, we were also not able to verify our third hypothesis, namely that autistic traits would moderate the effect of HR feedback on affective ratings. However, we found a significant three-way interaction of autistic traits, HR feedback, and target person, showing that the effect of the HR feedback on other ratings seems to be weaker for participants with higher AQ scores. Further, our results can be interpreted in such way that these participants differentiated less between self and other ratings. In the following, we will discuss these findings in detail and outline how our results can be informative for the discussion on the underlying mechanism on mindreading.

Our analyses showed that participants used the provided information, namely the HR feedback, differently for self and other ratings. During accelerated HR feedback trials, participants rated their gambling partner as being in a more negative state compared to their self-referential judgements, suggesting different processes for self- and other-directed affective mindreading. Two possible explanations for this finding arise:

First, different information might have been used during self- and other-directed mindreading: When judging the gambling partner, participants might have relied on the external sensory signal we provided, i.e., the accelerated HR feedback, likely because it was the only psycho-physiological information they had about them. Several participants indicated in our qualitative follow-up questionnaire that they indeed tried to interpret the HR signal to infer their gambling partner’s mental state. During self trials however, participants might have relied on additional own sensory signals, e.g. their actual heart rate, but also other cues such as their blood pressure^36,37^ or their own facial expressions (cf. facial feedback hypothesis^38^). It may also be that participants preferred their own sensory signal (i.e., their real HR) to the external signal we provided (the acoustic HR feedback) because people often (unconsciously) weigh their internal, unobservable cues stronger than other available information (e.g., behavior^39^). This explanation can also be linked to recent theories of cognitive functioning of the human brain (e.g.,^40,41^). For instance, the own interoceptive signal (the actual HR) and the external signal (HR feedback) could have differed in their precision-weighting^42^. The internal signal, i.e., the actual HR, might have been assigned to a higher precision for self-referential judgements, thus resulting in a stronger influence on the judgment than the external HR feedback. Previous studies already showed that internal signals related to the heart beat influence human cognition subconsciously^43^, a phenomenon that also fueled the discourse of interoceptive inference^42^. Embedding our results in the ISA theory^5^, our data do not rule out one common underlying mechanism to mindreading in self and others, namely the interpretation of sensory stimuli (including higher concepts bound to these stimuli). Rather, the weighting of the interpreted information for self and others might differ, thus leading to potentially different evaluations in self- and other-directed mindreading.

A possible second explanation why only ratings concerning the gambling partner were affected by the HR feedback is based on findings regarding a self-enhancement bias in affective judgements^26^. Previous study results indicated that participants judge themselves as being in a more positive state compared to (hypothetical) others. This bias diminished when the social distance between self and others was reduced. We tried to control for this bias through inducing high levels of felt similarity using a minimal group paradigm. We assumed that this hindered the bias during the control HR feedback, but it might still be possible that during accelerated HR feedback trials, the human tendency of self-enhancement might have prevailed. Participants might have used the accelerated HR feedback in other trials as an unconscious explanation to judge their gambling partner as being in a more negative affective state compared to themselves. Such a context-specific enhancement of biases, for example through induction of stress, has been demonstrated before^44^. Taken together, we propose that the distinction between self and other ratings in accelerated feedback trials is an indicator that not only outcome and expectancy values were used for the affective judgements, but that indeed subjects sought to mindread as they inferred different states for themselves and their gambling partner.

Our second significant finding is that the differential effect for self and other ratings during accelerated HR feedback trials was moderated by autistic traits. This three-way interaction bears two aspects: First, with increasing AQ scores, the differences between self and other ratings seem to diminish. Second, while it appears that individuals with lower AQ scores used the accelerated HR feedback as an informational cue, at least when judging their gambling partner, individuals with higher AQ scores seem not to have relied on this information, neither for self nor for other ratings. This finding could be interpreted as indication that participants with higher AQ scores might use different information (or a different weighting of information) while mindreading. This potential usage of different information might explain previous study results which suggest general differences in mindreading between autistic and non-autistic individuals^13,17,45^. Our results can be interpreted in a way that participants with high autistic traits mainly focused on clear unequivocal information, such as the gambling outcome or the expectancy value, in our paradigm. However, the ambiguous information of HR feedback seems to have been neglected for self and other judgements. Additionally, not only the ambiguity of stimuli but also the fact that we feedbacked an originally interoceptive signal (the HR) might have led participants with higher autistic traits to neglect this information during mindreading. Various studies reported altered interoception in autism, likely due to atypical sensory processing within this condition (for a review see^46^). Accordingly, it could be that for participants with high autistic traits, (originally) interoceptive signals are categorized as rather unreliable and will therefore not be interpreted—regardless of whether the access to this information stems from interoceptive or exteroceptive channels. In line with this interpretation, it was hypothesized that autistic individuals might trust their “gut feeling” (p. 409^47^) less and rely on explicit reasoning instead. Again, this can be incorporated into our hypothesis of different information weighting during mindreading. In our paradigm, sensory information might be weighted as less reliable information for mindreading in individuals with high autistic traits and thus be disregarded. Following the idea of precision weighting, this might also explain why we found the significant three-way-interaction suggesting there was a reduced differentiation between self and other judgements for individuals with high autistic traits: Self- and other-related stimuli might have been assigned with the same precision, thus leading to similar judgements. It was shown before that individuals with high autistic traits differentiate less between self and others^22^ and results of a self-other visual perspective taking task even lend the notion to the existence of a self-other intrusion in autism^48^. Taken together, we speculate that a common information weighting for self- and other-related judgements could explain our and previous findings of a reduced self-other distinction in individuals with high autistic traits (for a discussion on self-other distinction in autism see^49^). Further, we hypothesize that differences in information weighting might explain the often described differences in mindreading between autistic and non-autistic individuals. If mindreading alterations in autism are indeed a consequence of different information weighting, for instance social misunderstandings might be attenuated by giving additional, unequivocal information. Furthermore, if mindreading alterations equally concern the inference of own mental states in autism, (therapeutical) techniques originally developed to understand mental states of others could also be used for self-referential judgements.

Contradicting our expectations, we did not find that the HR feedback generally intensified affective ratings. We based our hypothesis on previous findings where neutral face stimuli were rated as more intense during accelerated HR feedback^27^. Our results show that participants judged their gambling partner as being in a more negative state in accelerated HR feedback trials across all outcome conditions. We already outlined that it appears the HR feedback was only used for the gambling partner ratings, whereas self ratings might have been based on own (interoceptive) information. In addition, our participants seemed to have interpreted the accelerated HR not as a sign of intensity of the gambling partner’s emotion but as an expression of negativity. Recent findings showed that a fast HR feedback induced an interoceptive illusion of effort in a cycling task whereas the feedback of a slower HR did not alter participants judgements^50^. In the context of our study, we suggest that participants associated the accelerated HR feedback with (emotional) strain, and thus interpreted the emotional states of their gambling partners as more negative. A biological underpinning to this hypothesis can be based on the link between HR signal and negative states such as anxiety and fear (for an overview see^51^).

### Limitations, strengths, and future directions

We acknowledge that we tested a very narrow interpretation of Carruthers’ interpretive sensory-access account^4^, i.e., identical processes in self and other-directed mindreading, but in our view such rigid interpretation lends itself best to test this one-mechanism account in the context of a mere behavioral set-up. Another limitation of our study is that participants did not listen to an acoustic feedback representing their real current HR but only to an approximation thereof. Thus, we cannot verify that the operationalization of the accelerated HR signal has worked as intended for all participants. We tried to address this potential problem through excluding participants that had higher HR baseline values in the second part of the experiment compared to their accelerated HR in the first part. Despite the exclusion of these participants, we acknowledge that the operationalization might not have worked in all trials. We are aware that HR is very sensitive and dynamic with regard to the current context of a person and that therefore also our HR feedback in control trials might not have represented the real sensory states of our participants. In general, we recognize that participants’ beliefs about the study’s manipulations and cover-story are relevant for the interpretation of the results. However, using the information of our qualitative follow-up questionnaire, we assume that most participants engaged in the wheel of fortune gamble, tried to infer their partner’s mental state, and believed in the HR manipulations. Another limiting aspect of our study is that we combined ratings of emotional valence and arousal within one scale. Although it might have been interesting to analyze valence and arousal separately, we decided to use a combined scale to reduce the number of evaluations, thus facilitating spontaneous affective mindreading and reducing habituation of emotional reactions to gambling outcomes. We recognize that separate scales might have unraveled more fine-grained processes, but we assume that this might have come to the cost of a lower validity of our paradigm. Regarding the composition of our sample, we did not specifically target a specific gender distribution. Due to the already complex design of our paradigm (3 × 2×2 repeated measures design), we refrained from adding gender as an additional variable in our study. However, we acknowledge that gender effects can play a crucial effect in mindreading performance^52^ and should be investigated further. We think, that due to the naturalistic set-up of our paradigm, it might be well suited to test gender effects in affective mindreading. A further limitation comes from research which indicates that co-occurring alexithymia is responsible for a large degree of the heterogeneity within the ASD population, particularly within the emotional domain (c.f. “alexithymia hypothesis”^53^). Previous research suggested that whenever emotional processes in autism are investigated, levels of alexithymia should be considered as they might be the cause to differences within these domains^53^. Alexithymia is a subclinical trait characterized through difficulties in identifying and/or describing one’s own feelings^54^ and was also linked to impaired interoception in autistic participants^55^. Therefore, our study results might also be related to the individual levels of alexithymia. Future studies should thus investigate the role of alexithymia in context of the ISA.

Clearly, as we could not verify our original hypotheses, our results have to be interpreted with care as they are post-hoc interpretations of the significant statistical effects within our model. However, an important goal of this study was to create a new experimental set-up that induces different affective states in a comparable self and other setting. Our results show that we were able to establish such a context as all our different outcome scenarios were rated significantly different in the respective valence direction. Although induction of affective states may seem trivial, experimental settings often face problems in inducing different affective states^56–58^. Furthermore, there are almost no studies that were able to validly compare self and other ratings in a comparable set-up. As we tested two real participants at the same time, and not merely confederates (e.g.,^59^) or video recordings (e.g.,^60^), we assume that our participants were more motivated to engage in affective mindreading. In addition, we were able to target current mental states, unlikely biased by memory or other processes.

We deem our study as a fruitful start for future research within this domain and important insights can be derived through the study of autistic participants. Based on our results, we assume that differences between autistic and non-autistic participants will emerge regarding which information they use to infer own and others’ mental states. Future studies could enhance the intensity of affective states for participants by increasing the subjective value of the gambling outcomes through, for instance, higher monetary compensations. Also, the characteristics of the stimuli that are (supposedly) interpreted during (affective) mindreading should be investigated further. In this paper we give two possible explanations why the HR feedback was neglected by participants with high autistic traits. On the one hand, it might be due to the weighting of internal in contrast to external signals. Future studies could test this hypothesis using the framework of predictive processing^40,41^ and different precision weighting. On the other hand, the effect could be attributed to the ambiguity of the HR feedback. In general, future studies should focus on the specific relationship of autistic traits on experimental manipulations, using more specific and continuous measures. We hope that future studies will address these issues to advance our understanding of the underlying mechanism to self- and other-directed mindreading in non-autistic and autistic individuals.

## Summary and conclusion

Based on theories suggesting a common underlying mechanism to mindreading in self and others, we developed a set-up to test this assumption. As mindreading was often reported to be altered in autism, we were interested in which way autistic traits would influence these mental state inferences. We used a simultaneous self- other gambling set-up and investigated three main research questions: First, we were interested if the same information is used to infer own and a gambling partner’s mental states. Our moderation analyses revealed that the experimental sensory information was used differently for self and other ratings, thus arguing against identical processes in self- and other-directed affective mindreading in our paradigm.

Second, inspired by the ISA account^4^, we were interested if mindreading is indirect and an interpretation of sensory information. Our results partly confirm this assumption: On the one hand, the accelerated HR feedback we provided was used to judge the gambling partner’s affective state. On the other, this sensory information was most likely not interpreted during self-referential judgements. We hypothesize that participants might have interpreted their own real HR or other internal signals during self-trials.

Third, we were interested if the level of autistic traits would influence the interpretation of sensory information. Our results can be interpreted in favor of this assumption: Although we were not able to detect the hypothesized effect of intensified affective judgements, autistic traits modulated the effect of HR feedback on gambling partner ratings. Our results suggest that individuals with high autistic traits did not interpret the HR signal when judging others (as compared to individuals with low autistic traits) and differentiated less between self and other ratings.

Taken together, even though the results of our study cannot fully support the idea of the ISA, it is possible that a common underlying mechanism, namely the interpretation and weighting of sensory information, might be at work during mindreading in self and others. Interestingly, the result that participants with high autistic traits differentiated less between self and gambling partner judgements fits to the notion of one-mechanism theories. We assume that individuals with high autistic traits might rely less on (external) sensory information when inferring mental states. A possible explanation could be a different weighting of information that might also explain often reported differences in mindreading between autistic and non-autistic participants. To conclude, we think that especially the study of autistic participants can shed further light into the debate on mindreading in self and others and their underlying (common) mechanism.

## Methods

### Participants

Based on previous studies^61–63^ and a sample size calculation using the interaction effect described in^27^, we recruited 59 non-autistic participants via online advertisements and an inhouse participant database. Two participants were excluded from analyses because of incomplete and noisy data. In addition, seven subjects were excluded due to characteristics of their HR measurement (see below) leading to a finale sample size of 50 participants (26 female, *m*_age_ = 30.04 years, *range* = 21- 42, *SD* = 5.1). All participants were fluent in German and did not use any psychoactive medication. The study was designed in adherence to the declaration of Helsinki and approved by the ethics committee of the Humboldt-Universität zu Berlin. All participants gave their written informed consent and were reimbursed with ten Euros.

### Stimuli

#### Wheels of fortune

The self-other gambling paradigm was inspired by previous studies^25,26^ during which participants rated their own or a fictive partner’s affective state after seeing the outcome of a wheel of fortune (see Fig. 1). The wheels of fortune were composed of three different segments, either indicating a *positive* (+ 5 Cents), a *negative* (-5 Cents), or a *neutral* (0 Cents) outcome. The different outcomes were color coded (positive = green, negative = red, neutral = grey) to facilitate outcome recognition. We designed 12 different wheels with varying segment sizes to accommodate nine different theoretical expectancy values. We used this operationalization to increase the variability of affective states and to make the experiment less simplistic as participants should combine information on the outcome and the expectancy value for each trial (e.g., affective states should differ between winning trials with a low vs high expectancy value). The expectancy values were calculated for each wheel as the sum of the respective sector sizes times sector values (0, + 5, − 5). We will refer to this factor as “Expectancy Value”.

#### Heart rate feedback

During the whole paradigm we presented an acoustic signal that was supposed to represent the participant’s (during self trials) or their gambling partner’s (during other trials) heart rate (HR) via headphones. A similar operationalization was repeatedly used in studies that aimed to feedback cardiac sensory information^27–29^. As a cover story, we told participants that the experiment was a pilot session for a planned fMRI experiment using the same paradigm. We explained that participants in fMRI studies are obliged to wear headphones for safety reasons and that (due to the pressure on the auricles) a common side effect in these studies was that participants would hear their own heartbeat. To control for this effect in our study, we would therefore feedback the participant’s own HR in self trials. In other trials, however, we would feedback the HR of their gambling partner to keep the effects of HR comparable between self and other ratings. Unknown to our participants, we feedbacked the same HR in self and other trials and accelerated the HR signal by 10% in half of the trials. We expected that participants would interpret the accelerated signal as an indicator of emotional intensity similarly in self and other trials, thus supporting the notions of the interpretive sensory-access account.

As we could not feedback the real time HR due to constraints of the utilized software, we used an approximation of the participants’ HR. Therefore, we recorded a two-minute baseline measurement with ECG electrodes (Zephyr©) placed in chest straps (Zephyr©) while participants were selecting pictures as part of the minimal group paradigm, directly prior to the wheel of fortune gamble. We then computed an average beat per minute (bpm) and acoustically feedbacked this beat during the *control* HR condition. For *accelerated* feedback trials, we accelerated the baseline HR by 10%. For the second half of the experiment, we repeated this procedure with a new two-minute baseline measurement that we recorded during the break. We used two baseline measurements as we expected the heart rate to slow down with time. For the whole experiment, participants believed that they would listen to their (or their gambling partner’s) current HR. This means, participants were unaware about the averaged nature of the feedback, about the manipulation during accelerated feedback trials, and about the fact that they listened to the same signal in self and other trials.

#### Autism-Spectrum Quotient

We used the Autism-Spectrum Quotient (AQ^30^) to assess the broader autistic phenotype in non-autistic adults. This questionnaire consists of 50 items targeting the five different domains social skills, attention switching, communication, imagination, and attention to detail^30^. Higher autistic traits are indicated through higher AQ scores with a clinically meaningful cut-off at 32 (range = 0 to 50).

#### Self-other gambling paradigm

**Figure 3:**
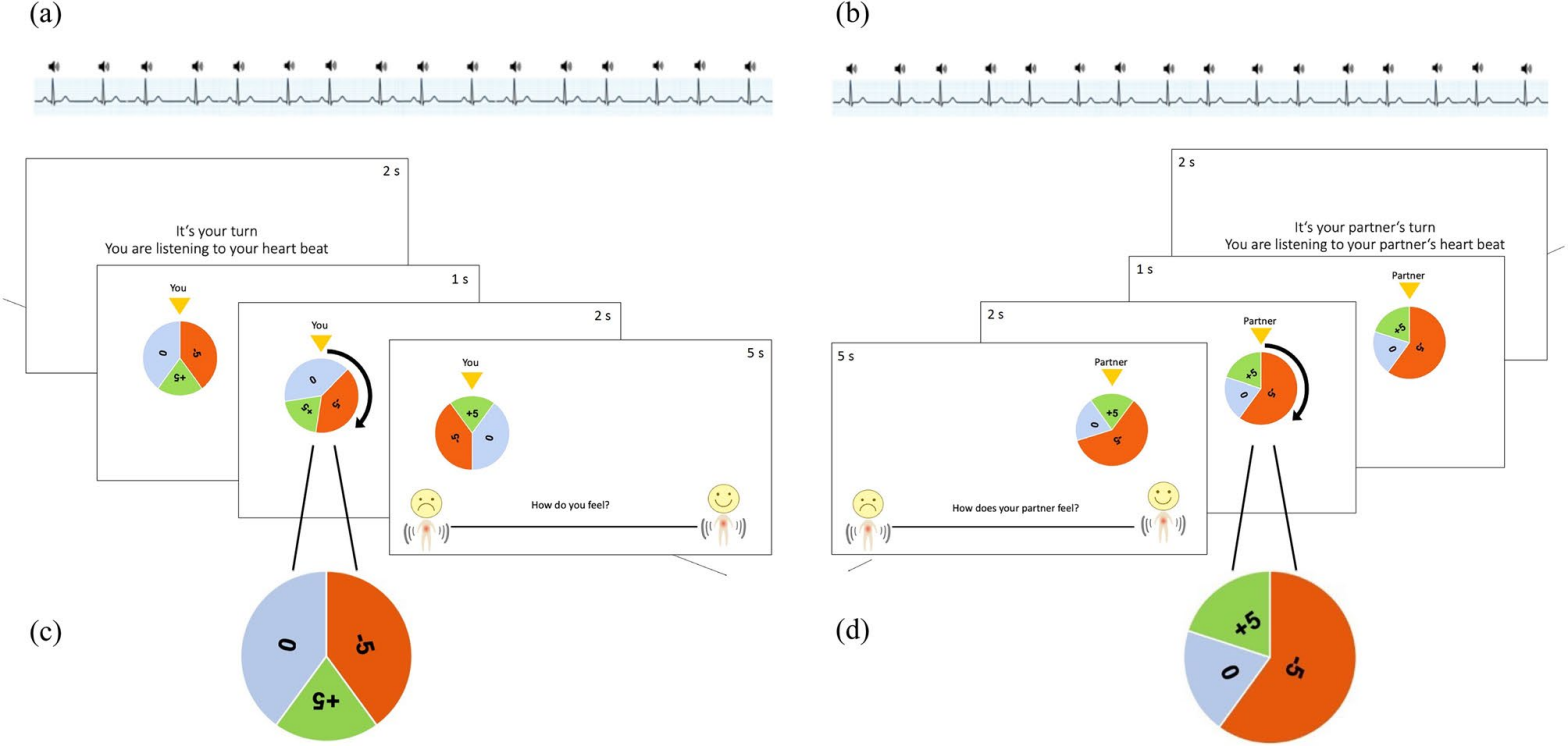
Depiction of the self-other gambling paradigm. *Note* An exemplary self trial is depicted on the left (**a**) and an exemplary other trial is depicted on the right (**b**). Different Expectancy Values in these trials are illustrated at the bottom (see augmentations depicted in (**c** and **d**). As visualized on top of the figure, participants listened to an acoustic signal representing their or their gambling partner’s HR throughout all trials.

The paradigm was programmed in MATLAB^64^ using the Psychophysics Toolbox extensions^65–67^. The experiment was divided into 24 blocks à six trials with a short break after half of the trials. At the beginning of a block, participants were informed for which target person they would give ratings (*self* vs *other*). Then, one out of the 12 different wheels of fortune was presented for one second, rotated for two seconds, and stopped. A pointer indicated the outcome of the wheel and participants had five seconds to judge their own, or their gambling partner’s, affective mental state by clicking on a visual scale from 0 (very intensively negative) to 100 (very intensively positive). To enhance the affective involvement, we told participants that they and their gambling partner would receive the final sum of all their trials at the end of the experiment. We expected that the additional HR feedback would be interpreted predominantly as a manifestation of arousal and thus mixed both dimensions in one scale. Integrating arousal and valence in one scale rather than probing them differently reduced the judgments by half, which we deemed important to reduce habituation effects and keep motivation for mindreading high. An exemplary self and other trial are depicted in Fig. 3. In every block, each outcome (positive/negative/neutral) occurred at least once. Over all trials, the different outcome trials added up to 0 Cents in the end. In total, participants rated 144 trials in 12 self and 12 other blocks. Out of these 12 self/other blocks, six were presented with a control and six with an accelerated HR. Our experimental paradigm was a 3 (Outcome) × 2 (Target Person) × 2 (HR Feedback) repeated measures design. The 24 experimental blocks were presented in a pseudo-randomized order (not more than two blocks of the same condition were presented subsequently). Apart from this restriction, the block order was chosen randomly for each person to attenuate block order effects.

To foster a need for mentalizing, i.e., to increase the chance that participants took the perspective of their gambling partner in other trials—and not just reported their own current state—we told participants that the ratings of both gambling partners would be recorded and compared instantly. Therefore, participants believed that if their judgements regarding their gambling partner’s mental states and their gambling partner’s judgment about their own mental states were close enough, we would reward them with additional money at the end of the experiment. All participants were rewarded with two Euros in addition to the originally advertised eight euros to compensate the facts that we did not compare the ratings between both partners and that there was no financial gain in the gamble as all outcomes equaled out to zero.

### Procedure

Prior to the testing session, we sent out an online questionnaire including demographic questions and the AQ^30^ to assess the level of autistic traits. For the testing day, we invited two participants unknown to each other to play an allegedly interactive gamble. As described, their main task was to infer and rate, respectively, the own and the gambling partner’s current affective states after seeing the outcomes of different wheel of fortune trials. In addition, participants were listening to an acoustic signal representing their own or their gambling partner’s heart rate. In the few cases where one participant had to cancel the session, a confederate experimenter pretended to be the second participant. Both participants were shortly introduced to each other and tested in two adjacent rooms with an identical experimental setup. Throughout the whole experiment participants were made to believe they played the game simultaneously together with their gambling partner.

As previous research has demonstrated that felt similarity increases emotion attribution^26^, we manipulated felt similarity between participants through a minimal group paradigm^68^. It was shown before that participants attribute more similar subjective experiences to members of their ingroup compared to those of an outgroup^69^. To induce feelings of similarity, we presented five pairs of one impressionistic and one expressionistic artwork and asked participants to indicate their preference for each pair. We summed up the overall preference for expressionistic and impressionistic pictures and feedbacked that the participant in the other room had the same art preference. Through this, we tried to induce a comparable degree of felt similarity for all participant pairs. Subsequently, we started the self-other gambling paradigm including the HR feedback. The experiment started with two practice blocks (one for self and one for other ratings), which was followed by 24 experimental blocks. Once participants finished the experiment, we administered a qualitative follow-up questionnaire that included questions on how much money participants thought they had won during the experiment, how similar participants would rate themselves and their partner (0–100%), and if they had noticed anything particular during the experiment. After the participants had filled out the questionnaire, we debriefed participants and told them about the manipulations and the purpose of the paradigm.

### Statistical analyses

All analyses were conducted with the statistical programming software R^70^. To examine if our HR manipulation had worked as intended, namely that the accelerated HR feedback was faster than the actual HR of our participants, we compared the control and accelerated HR for each subject. Our reasoning was as follows: Since we did not measure HR continuously of participants, we used the two baseline measurements for determining the frequency of the feedback. As described above, we took two baseline measurements because we expected the heart rate to slow down with time. If our second baseline measurement, i.e., the control HR in the second half of the experiment (e.g., 70 bpm), was higher than our manipulated accelerated HR signal in the first half of the experiment (e.g., 66 bpm), we could not rule out that our accelerated feedback manipulation had failed (because we expected the HR to slow down with time). For this reason, we excluded seven participants. We used linear mixed effects models (lmms) with sum contrast coding and maximum likelihood estimation to evaluate our three hypotheses (estimated with the nlme package v3.1–139^71^). We modelled the variables participants and stimulus (i.e., the 12 different wheels of fortune) as random intercepts. Further we incorporated a random slope for the factor Outcome.

Our first hypothesis targeted the assumption of one-mechanism theories, namely that self- and other-directed mindreading share a common underlying mechanism. Therefore, given the similarity of the self and other conditions, we hypothesized that the same information would be interpreted in self- and other-directed mindreading trials and no differential effects for the factor Target Person (self vs other) would emerge. We tested this hypothesis using model comparisons (see supplementary table S1 for all models). We started with a theoretical baseline model that incorporated all described variables as main effects—except the variable Target Person—as well as the hypothesized interaction of Outcome*HR Feedback and the three-way interaction of Outcome*HR Feedback*AQ. In principle, our baseline model represented the notion of identical processes in self- and other-directed mindreading trials. After comparing the baseline model to a model including Target Person as an additional main effect, we subsequently included moderation effects, i.e., an additional interaction term accounting for differential effects between self and other ratings, for each predefined main and interaction effect. If self- and other-directed mindreading were indeed identical processes in our paradigm, there should be no model fit improvement by adding the factor Target Person as main or interaction effect. To evaluate model fit, we used the information criteria AIC and BIC. Models with lower values of these fit indices indicate better fit of the model^72^. In case of conflicting results, i.e., AIC favoring one and BIC favoring another model, we performed additional likelihood ratio tests. Comparing baseline model to moderation model, we chose the one that indicated better model fit. This model was then used as basis for the next comparison.

To test our second hypothesis, that mindreading is indirect and an interpretation of sensory information, we investigated the interaction of the factors Outcome and HR Feedback. We assumed that during accelerated HR Feedback trials, ratings for negative outcomes would become more negative (closer to 0) and ratings for positive outcomes more positive (closer to 100). We based this hypothesis on the idea that participants would interpret the HR signal as an indicator of arousal and thus infer an intensified affective state.

Our last hypothesis targeted autistic traits. We hypothesized that individuals with higher AQ scores would use the HR Feedback to a lesser extent than individuals with low AQ scores. Therefore, we tested the three-way interaction Outcome*HR Feedback*AQ.

### Ethical approval

The study protocol was reviewed and approved by the ethics committee of the Department of Psychology at Humboldt-Universität zu Berlin. The study was conducted in accordance with the Declaration of Helsinki.

## Data Availability

Data (excluding demographic information) and code of the model comparisons are openly available at the OSF repository: https://osf.io/3q2ub/.
